# Representative Cell Analysis for Damage-Based Failure Model of Polymer Hexagonal Honeycomb Structure under the Out-of-Plane Loadings

**DOI:** 10.3390/polym13010052

**Published:** 2020-12-25

**Authors:** Muhammad Salman Khan, Ainullotfi Abdul-Latif, Seyed Saeid Rahimian Koloor, Michal Petrů, Mohd Nasir Tamin

**Affiliations:** 1 School of Mechanical Engineering, Faculty of Engineering, Universiti Teknologi Malaysia, Johor Bahru 81310, Malaysia; engrsalman091@gmail.com (M.S.K.); ainullotfi@utm.my (A.A.-L.); 2 Institute for Nanomaterials, Advanced Technologies and Innovation (CXI), Technical University of Liberec (TUL), Studentska 2, 461-17 Liberec, Czech Republic; s.s.r.koloor@gmail.com (S.S.R.K.); michal.petru@tul.cz (M.P.); 3 Department of Aerospace Engineering, Faculty of Engineering, Universiti Putra Malaysia, Serdang 43400, Malaysia

**Keywords:** finite element simulation, Hashin damage criteria, polymer hexagonal honeycomb core, out-of-plane behavior, representative cell model

## Abstract

The honeycomb (HC) core of sandwich structures undergoes flexural loading and carries the normal compression and shear. The mechanical properties and deformation response of the core need to be established for the design requirements. In this respect, this article describes the development of the smallest possible representative cell (RC) models for quantifying the deformation and failure process of the Nomex polymer-based hexagonal HC core structure under the out-of-plane quasi-static loadings. While the hexagonal single and multi-cell models are suitable for the tension and compression, a six-cell model is the simplest RC model developed for shear in the transverse and ribbon direction. Hashin’s matrix and fiber damage equations are employed in simulating the failure process of the orthotropic cell walls, using the finite element (FE) analysis. The FE-calculated load–displacement curves are validated with the comparable measured responses throughout the loading to failure. The location of the fracture plane of the critical cell wall in the out-of-plane tension case is well predicted. The wrinkling of the cell walls, leading to the structural buckling of the HC core specimen in the compression test, compares well with the observed failure mechanisms. In addition, the observed localized buckling of the cell wall by the induced compressive stress during the out-of-plane shear in both the transverse and ribbon direction is explained. The mesoscale RC models of the polymer hexagonal HC core structure have adequately demonstrated the ability to predict the mechanics of deformation and the mechanisms of failure.

## 1. Introduction

Honeycomb (HC) sandwich panels have found numerous engineering applications, such as wind turbine blades, aircraft wings, spoilers and engine cowls, yacht hulls and floor panels, and surfboards. The HC sandwich panels exhibit a high strength-to-weight ratio, high structural stiffness, and improved resistance to the harsh operating environment. In addition, these lightweight structural materials offer an excellent capability to withstand through-thickness compression. The HC sandwich panel is constructed by laminating the outer surfaces of a HC cellular core structure with thin and stiff face sheets. It is designed such that the HC core not only maintains the distance between the face sheets and improves the flexural stiffness but also carries the normal compression and shear loads [1]. The common HC cores with square or hexagonal cells [2] are fabricated from metallic alloys such as aluminum [3,4], and polymers including Kevlar or Aramid resin-impregnated papers [5,6]. Recent advances in 3D printing technology have made it easier to fabricate cellular core structures from other polymeric materials such polylactic acid (PLA) [7,8] and Acrylonitrile Butadiene Styrene (ABS) [9]. The face sheets are typically made of aluminum, glass fiber-reinforced polymer (GFRP), or carbon fiber-reinforced polymer (CFRP) composite laminates. The HC sandwich structures are often subjected to complex operating loads, including the in-plane and out-of-plane loadings. Depending on the geometric specifications, the HC core exhibits an anisotropic response under the quasi-static and low impact loading conditions [10]. A thorough understanding of the mechanical responses of the polymeric HC core is thus inevitable in quantifying the performance and reliability of the HC sandwich structures.

Analytical models employing the ideal hexagonal cell geometries have been developed [11,12,13]. These models provide the first approximation of the elastic deformations of the HC core structure and the stress fields in the cell walls. The numerical modeling of the HC core structural behavior under general loading conditions has been performed at both macro- and meso/micro-length scale. The macro-mechanical approach employs an equivalent homogeneous solid. The model does not account for the localized buckling of the core [14,15]. The meso/micro-mechanical model utilizes the representative unit cell of the HC core structure [16,17,18]. The unit cell could consist of a single or multiple number of connected cells. The models account for the details of the geometric features of the cellular structure and the cell wall material. Consequently, such a unit cell model could accurately simulate the deformation and fracture processes of the HC core. The use of continuum (3D) elements further improve the accuracy of the finite element (FE)-calculated responses over the more computationally efficient conventional shell (2D) elements [15]. An equivalent spring element model has also been used for modeling the flatwise compression response of HC core [11,19]. The success of the abovementioned models relies, to a great extent, on the availability of the experimentally-determined structural and material properties of the HC cores.

**Figure 1:**
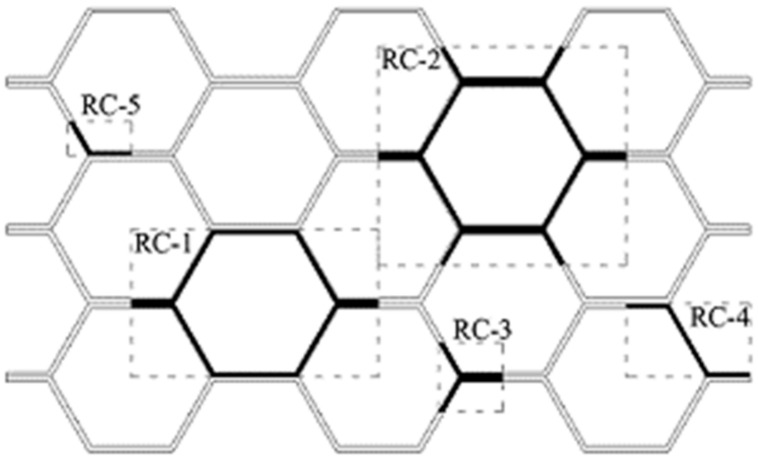
Representative unit cell (RC) models for hexagonal honeycomb (HC) core.

Unit cell models should represent the true response of the HC core structure under the applied loading conditions. The model takes the cell wall material properties and predicts the structural properties and behavior of the HC core panel with multiple cells. The Nomex polymer-based HC core (HRH-10) cell wall, made of fiber-reinforced aramid papers with phenolic resin coating, has been modeled as a single-layer isotropic [12,13,16], single-layer orthotropic [6,11,15,20], and multi-layer resin coating [21,22,23] material. The single-layer orthotropic material is the most common and provides greater flexibility in specifying directional mechanical behavior [24]. Works on different representative unit cell models, as illustrated in Figure 1 have been published. Liu et al. [22] and Pan et al. [25] used the unit cell model consisting of a half double cell wall and part of the adjacent single walls (RC-3) in predicting the out-of-plane behavior of polymeric Nomex HC core structures. It was found that de-bonding at the double-wall edges is the most critical for buckling to occur [22]. Becker [17] incorporated a single inclined wall and half of the adjacent double walls (RC-4) to predict the in-plane stiffness of the HC core. The derived results show that the core thickness has a significant effect on the mechanical properties and therefore, needs to be accounted appropriately. The boundary conditions for these unit cell models are more complex than those consisting of a single-cell or multiple closed cells. The unit cell model with a hexagonal cell and part of the single and double-wall at the corners (RC-2) could provide a realistic simulation of the cell wall folding mechanism under the out-of-plane compression [12,24]. However, the model overestimates the structural properties of the HC core under the compressive loading [24]. A more relatable unit cell model comprising of a hexagonal cell and part of the double-wall edges on both sides (RC-1) is implemented by many researchers. The predicted elastic properties are in good approximation with the measured test data for out-of-plane loading conditions [26,27,28]. The numerical results also illustrated the brittle response of the polymer resin-impregnated Nomex paper for HC core, compared to aluminum which resulted in a higher critical buckling load [27].

Buckling failure of the sandwich structures under the flatwise compression has been observed to initiate and localized in the HC cores [11,29]. In addition, failure also initiated in the HC core at the core/face sheet interface of the sandwich structures under the flexural loading [8,16]. The observed localized failure initiation leading to the catastrophic fracture of the sandwich structure necessitates the simulation of the complete failure process to capture the observed failure mechanisms. This calls for the constitutive model of the cell wall materials with appropriate failure criteria. In this respect, several failure criteria, including Hashin [30], Tsai-Wu [31], Tsai-Hill [32], and Sun [33], are of particular interest for an HC core made of unidirectional fiber-reinforced polymer papers. Jaafer et al. [34] employed Hashin failure criteria to model the fracture and optimize the cutting parameters in the machining process of the polymeric Nomex HC core. Yang et al. [35] used Tsai-Hill theory to investigate the strength of glass and carbon fiber-reinforced composite plates subjected to tensile and compressive load. Hashin failure criteria have also been employed in modeling the fiber-reinforced polymer composite laminates [36,37]. Giunta et al. [38] implemented the stress-based failure criteria known as Lee and Tsotsis criteria (LTC) [39] for characterizing the indentation behavior of the sandwich plates made of polymeric face sheets and foam core. The damage mechanics-based model enables the complete simulation of the elastic deformation through the fracture of cell wall material in a single formulation. Limited work is reported on the damage-based approach for quantifying the failure of the polymer Nomex HC core. In this respect, Ramirez et al. [40] proposed a decoupled modelling strategy for quantifying the buckling and collapse of the cell walls. However, the proposed damage model is implemented for the out-of-plane shear load case only. A recent study on the thermomechanical properties of composite sandwich panels emphasizes the need of a progressive damage model to quantify the various failure modes [41]. A comprehensive review on the equivalent model approaches is provided by Aborehab et al. [42]. The authors suggested that the shell–volume–shell (SVS) approach could be efficiently used for the macro-mechanical model of the sandwich panels having the face sheets modelled as shell element while the HC core as solid element.

Based on the reviewed literature above, the meso-scale FE models of the HC core are commonly created using large number of cells to accurately capture the mechanical behavior. However, this compromises the computational efficiency in terms of cost and time. In this respect, an efficient and validated FE model of the polymeric HC core is invaluable in view of the ever-increasing complexity of the lightweight structural design and simulation. In addition, a damage model is needed to predict the structural strength and simulate the progressive failure of the polymeric HC core under the general loading conditions. In this respect, the current work establishes a systematic methodology to identify the smallest representative cell model of the polymeric hexagonal HC core. The out-of-plane load cases consists of tension, compression, and shear in both the ribbon and transverse direction. The FE-calculated behavior of the polymer cell wall material acknowledges the damage initiation event and the subsequent evolution of the damage to localized fracture/buckling of the HC core. The experimental validation of the representative cell models considers both aspects of the mechanics of materials and the observed mechanisms of failure. The calculated responses of the validated representative cell models serve in providing high-fidelity input property data for the equivalent homogenized HC core model of the large structures, under the general loading conditions.

## 2. Framework for FE Simulation of Damage and Failure of Honeycomb Core Structure

The prediction of the deformation and failure processes of a polymeric HC core structure should consider both aspects of the mechanics of deformation and the mechanisms of failure of the structure. The mechanics is quantified in terms of the internal states of strains, stresses, and damage of the cell wall materials that manifest through the load–displacement responses. The mechanisms represent the different modes of failure of the HC core structure. These aspects could be captured through the finite element (FE) simulation of the structural response. A representative cell model with appropriate boundary conditions and the material constitutive model are required to faithfully reproduce the structural responses of the HC core to loading. In this study, a stress-based damage criterion was employed, to trigger the initiation of the damage in the cell wall material, while the subsequent damage evolution was described by the dissipation-energy-based criterion. The FE models were then validated by using the measured load–displacement curves and the observed failure modes of the HC core specimen for each load case considered.

### 2.1. Representative Unit Cell Models

**Figure 2:**
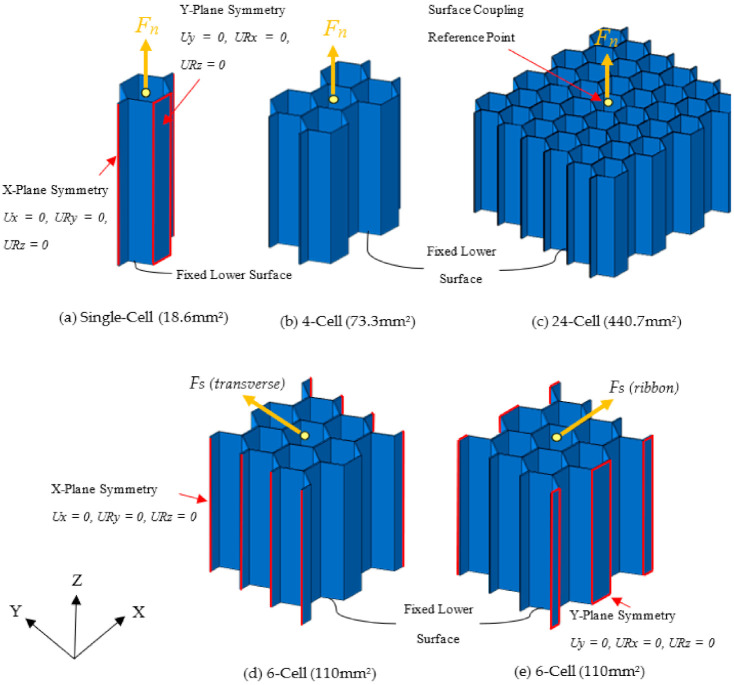
(**a**) Single-cell and (**b**,**c**) multi-cell models for the out-of-plane tension and compression, and (**d**,**e**) 6-cell model for the out-of-plane shear in the transverse and ribbon direction, respectively.

Several geometries of the representative unit cell of the HC core structure for the different out-of-plane loading and boundary conditions were examined in this study, as illustrated in Figure 2. A fixed size of the hexagonal cell (*c* = 3.2 mm) and cell height (*H* = 12.7 mm) were selected. Figure 2a represents the single-cell model made of one closed hexagonal cell with half double-wall on the two sides termed as RC-1 in Figure 1. It was employed for simulating the behavior of the out-of-plane tension and compression loading (in the *Z-*direction). In addition, the responses of the 4-cell and 24-cell models, illustrated in Figure 2b,c, respectively, for these load cases were also examined. The unit cell model with six closed cells and the half double-wall edges was dedicated for the out-of-plane shear load cases. The transverse shear load case consists of the applied shear force perpendicular to the double-walls orientation (*Y-*direction), while the ribbon direction refers to the shearing along the double-wall orientation (*X-*direction) of the hexagonal cells, as illustrated in Figure 2d,e, respectively.

The symmetry boundary condition is prescribed on the selected surfaces of the model, as indicated by the dark (red) colored areas in Figure 2. The symmetric boundary conditions mentioned for the single-cell model were also used in the 4-cell and 24-cell models, on the same respective double-wall surfaces and free edges. This boundary condition represents the displacement constraints imposed by the adjacent wall/cell. In the tensile and compressive load cases, the bottom section of the model is constrained in the *Z-*direction. A node located in the center of the bottom plane was fixed, to avoid the rigid body motion of the model. Iso-strain condition is prescribed for the top surface of the model, where the quasi-static displacement is applied in the +*Z* and −*Z-*direction for the out-of-plane tensile and compressive load case, respectively. In the out-of-plane shear load cases, the bottom surface of the model was fixed, while the top surface was subjected to the prescribed displacement in the *X-* and *Y-*direction for the ribbon and transverse shear load case, respectively. The *X*-plane symmetric boundary conditions are imposed on the free edges in the case of transverse shear loading, as shown in Figure 2d. For the shear loading in ribbon orientation, *Y*-plane symmetry was applied, to replicate the double-wall effect on the parallel walls, as given in Figure 2e. The adequacy of the boundary conditions in simulating the response of the HC core structure under the prescribed out-of-plane loading was thoroughly examined.

### 2.2. Materials, Properties, and Damage Models

**Figure A1:**
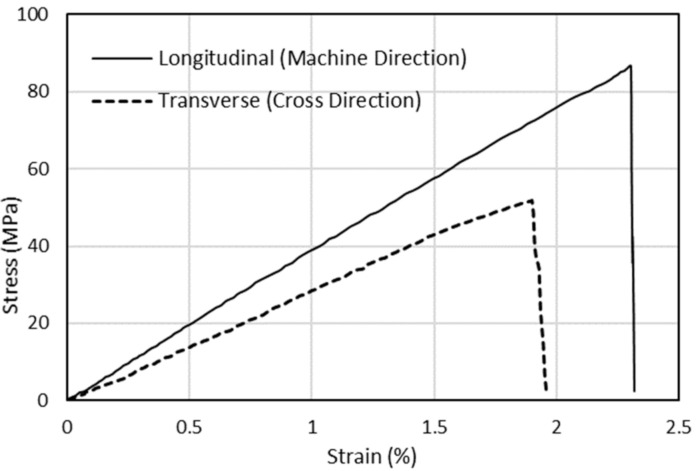
Tensile stress–strain curve of the Phenolic resin-based polymer Nomex paper.

The HC core panel used in this case study is based on the Nomex polymer-based paper. The cell wall is made of fiber-reinforced paper with phenolic resin coating. The tension tests of phenolic resin-based Nomex paper were conducted by employing similar mechanical test procedures as in Roy et al. [6]. The longitudinal direction reflects the tension load in the fiber orientation (0°), while the cross direction is for the transverse orientation (90°). A total of three specimens were tested for both of the orientations. The resulting stress–strain curves are presented in Appendix A Figure A1. The elastic property ($E_{11}$ and $E_{22}$) values were obtained from the respective test, while the remaining elastic constants were estimated based on the previous studies [20]. The fibers are primarily oriented in one of the in-plane principal directions in the polymer HC core, denoted as the 11-direction. Consequently, the thin cell wall is expected to behave as an orthotropic layer, with the material properties as shown in Table 1. It is noted that a slight anisotropy of the Nomex polymer-based paper is reported, as indicated by the 8% variation in the $E_{33}\;$ with respect to $E_{22}$. The polymer hexagonal HC core panel is constructed such that the principal fiber direction is aligned with the *x*-axis of the structure. This allows the HC core structure to have greater strength under the shear and flexural loading condition in that direction [43]. The resistance to buckling of the HC cells construction is of primary concern in the out-of-plane compression and shear loading of the polymer HC core.

**Figure 3:**
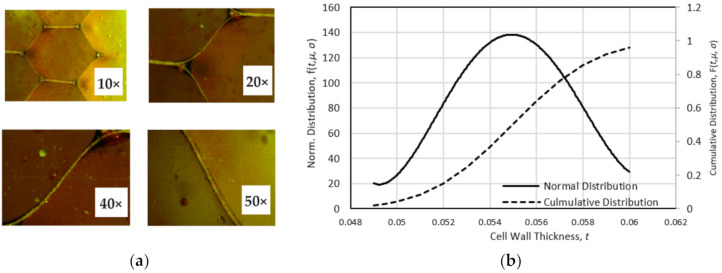
(**a**) Cell-wall thickness of the polymer hexagonal HC core at different magnification, and (**b**) distribution of the wall thickness of the HC cell.

It is worth noting that the property of the cell wall material, and thus the response of the polymer HC cell structure, is strongly dependent on the thickness of the cell wall [24]. This is derived from the relatively higher phenolic resin content in a thicker wall. The effects of the phenolic resin impregnation on the resulting properties of the paper and the cell behavior have been discussed by previous researchers [44]. In this respect, the statistical assessment of the variations of the wall thickness was performed. Thickness data of the cell wall were obtained by optical measurements at four different magnifications, as shown in Figure 3a. The resulting normal distribution of the thickness data is shown in Figure 3b. The mean wall thickness is 0.0553 mm, with one standard deviation of 0.0028 mm.

The failure process of the polymer hexagonal HC core structure is simulated within the continuum damage mechanics domain and at the meso-scale. The stress-relative displacement response of the cell wall material to failure is assumed to follow a bilinear softening law. Four potentially different damage mechanisms of the anisotropic cell wall material were considered. The stress-based damage initiation variable was computed for each damage mechanism, up to the onset of damage, as follows [30]:

Damage initiation criterion due to fiber fracture and buckling/kinking:

(1) $${\left(\frac{{\hat{\sigma }}_{11}}{X^{T}}\right)}^{2}+{\left(\frac{{\hat{\tau }}_{12}}{S^{L}}\right)}^{2}=d_{f}^{t};\;\;\mathrm{for}\;{\hat{\sigma }}_{11}\geq 0\;(\mathrm{Tension})$$

(2) $${\left(\frac{{\hat{\sigma }}_{11}}{X^{C}}\right)}^{2}=d_{f}^{c};\;\;\mathrm{for}\;{\hat{\sigma }}_{11}<0\;(\mathrm{Compression})$$

Damage initiation due to matrix cracking and crushing:

(3) $${\left(\frac{{\hat{\sigma }}_{22}}{Y^{T}}\right)}^{2}+{\left(\frac{{\hat{\tau }}_{12}}{S^{L}}\right)}^{2}\;=d_{m}^{t};\;\;\mathrm{for}\;{\hat{\sigma }}_{22}\geq 0\;(\mathrm{Tension})\;$$

(4) $${\left(\frac{{\hat{\sigma }}_{22}}{2S^{T}}\right)}^{2}+\;\left[{\left(\frac{Y^{C}}{2S^{T}}\right)}^{2}-1\right]\left(\frac{{\hat{\sigma }}_{22}}{Y^{C}}\right)\;+\;{\left(\frac{{\hat{\tau }}_{12}}{S^{L}}\right)}^{2}=d_{m}^{c};\;\;\mathrm{for}\;{\hat{\sigma }}_{22}\;<0\;\left(\mathrm{Compression}\right)$$

In these equations, the matrix $\left[{\hat{\sigma }}_{ij}\right]$ represents the effective stress in a meso-scale lamina; the parameters $d_{f}^{t}$, $d_{f}^{c},$ $d_{m}^{t}$, and $d_{m}^{c}$ are the internal damage variable in the respective fiber and matrix phases. The parameters $X^{T}$, $Y^{T}$, $X^{C}$, $Y^{C}$, $S^{L}$, and $S^{T}$ are the strength properties of the orthotropic cell wall material. All of these property values are listed in Table 1, where the longitudinal and transverse tensile strength ($X^{T}$, $Y^{T}$) are taken from the stress–strain plots (given in Appendix A Figure A1) of the tension test conducted for phenolic resin impregnated polymer Nomex paper. Each stress-over-strength criterion with a value of unity denotes the initiation of the respective damage in the cell wall material, at the critical point.

Following the onset of damage, the critical material point in the cell wall of the polymer HC structure would suffer from the degradation of the strength and stiffness properties. In this work, the damage is assumed to evolve according to the equivalent dissipation energy model obtained from the effective stress-displacement relation for each respective failure mode [36]. The critical value of the equivalent dissipation energy, *G_C_* equals to the fracture energy in each independent fracture mode. The value of the fracture energies *G_XT_*, *G_XC_*, *G_YT_*, and *G_YC_*, listed in Table 1, are used to specify the softening behavior and the characteristic stress degradation leading to the final failure of the material points. The corresponding damage evolution criterion is given by the following [36]:

(5) $$d_{p}=\frac{\delta_{eq}^{f}\;\left(\delta_{eq}\;-\;\delta_{eq}^{0}\right)}{\delta_{eq}\left(\delta_{eq}^{f}\;-\;\delta_{eq}^{0}\right)},\;\mathrm{with}\;\;\delta_{eq}\geq \delta_{eq}^{0}$$

where $\delta_{eq}^{0}$ is the equivalent displacement corresponding to the damage initiation (i.e., $d_{p}=0$), and $\delta_{eq}^{f}$ is the displacement at the failure of the material point ($d_{p}$ = 1).

### 2.3. Finite Element Simulation of the Failure Process

**Figure 4:**
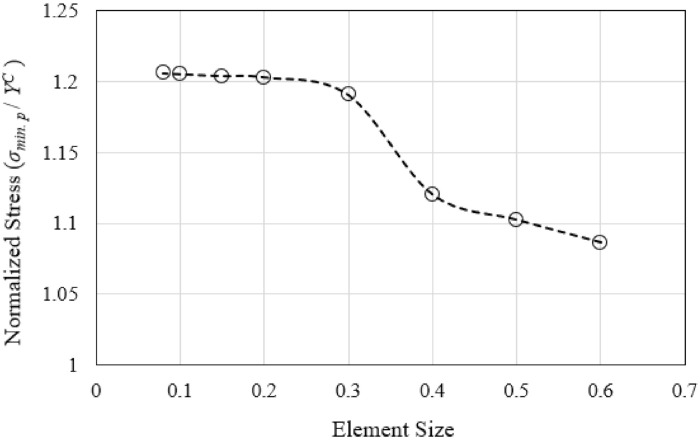
Mesh convergence analysis outcomes based on the out-of-plane compressive load case with the single-cell model.

The representative single-cell model for the out-of-plane compressive load case, as described in Section 2.1, was developed in ABAQUS FE analysis software (version 6.14.2) for the element mesh convergence analysis. The model was discretized into 8-node continuum shell elements (Abaqus SC8R element type). The analysis was performed to ensure that the FE-computed variables of interest were independent of the maximum element size employed. Successive runs, each with decreasing size of the element, were performed. The outcome of the analysis is shown in Figure 4 in terms of the normalized (minimum) principal stress corresponding to the applied displacement of 1.0 mm, as the monitoring variable. The minimum element size of 0.2 mm was then employed in subsequent analyses of all the load cases and the different geometry of the unit cell models. The single-cell model for the tensile and compressive load cases was discretized into 5402 elements, while the 6-cell model of the shear load cases consisted of 12,096 continuum shell elements.

**Figure 5:**
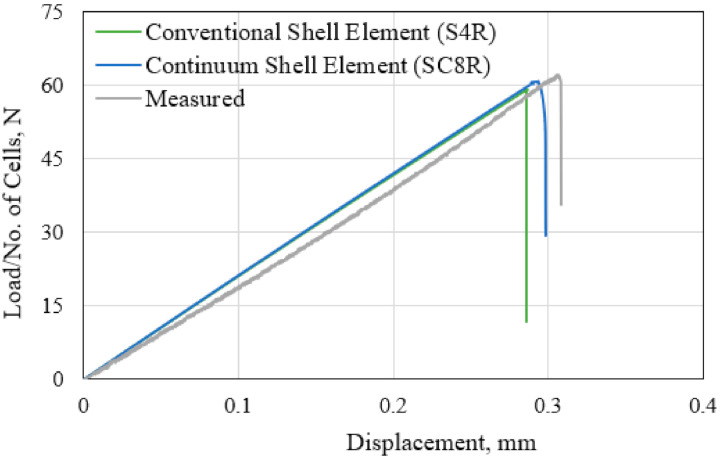
Comparison of the finite element (FE)-calculated responses of the single-cell model with the measured curve for the tensile-load case.

The computational efficiency of the FE simulation process is assessed by comparing the outcomes of simulating the tensile load case of the single-cell model. One model employs the conventional 4-node shell element (Abaqus S4R element type) which assumes a constant distribution of the calculated variables across the thickness of the cell wall. The other uses the continuum 8-node shell element (Abaqus SC8R element type). This element can capture the through-thickness variation of the stresses and damage responses. The simulation is performed using Intel^®^ Core™ i5 PC with 4 core processor, each having 2.8 GHz base frequency. Identical element topology and mesh size are used for both cases. The calculated load–displacement responses are compared in Figure 5, while the computational resources used are listed in Table 2. Results indicate that the continuum shell elements provide a closer prediction of failure to the measured response when compared to the conventional shell elements. However, the 17.2% larger number of the unknown variables resulted in a 63.6% longer wall clock time. In view of validating the proposed representative unit cell models against measured deformation, the continuum shell elements were used throughout the remaining load cases.

## 3. Results and Discussion

### 3.1. Out-of-Plane Tensile Response

**Figure 6:**
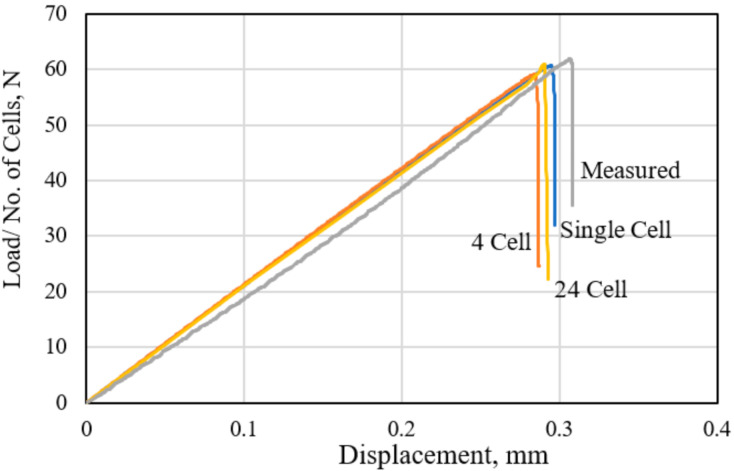
Comparison of FE-calculated and measured tensile responses of the HC core.

The FE-predicted load–displacement responses of the polymer hexagonal HC core models consisting the single-cell, four-cell, and 24-cell hexagonal models are compared with the measured response in Figure 6. The response-load values are normalized by the number of cells in each sample. The measured response is taken from the tests performed in accordance with the ASTM C297 standard, with the results presented in the previous research work [45]. The measured stiffness, as reflected in the slope of the *P-d* curve, is 5.1% lower than the calculated values. This is likely due to the inherent material inhomogeneity of the fiber-reinforced synthetic polymer-based cell walls. The observed sudden load drop at the peak value indicates the brittle fracture event. This brittle fracture mechanism is also reproduced by all the FE models of the HC core examined. However, the FE-calculated onset of the brittle fracture occurs slightly earlier than the observed fracture event. Such a damage-based prediction of the fracture event could be further improved by refining the element size in the damage process zone [36]. However, the peak load levels at fracture are comparable at 62.5 ± 0.5 N. Both the single and multiple cell models fairly reproduce the out-of-plane tensile responses of the HC core specimen. Consequently, the single-cell FE model, with the appropriate boundary conditions, is computationally efficient to accurately represent the out-of-plane tensile behavior of the hexagonal HC core panel fabricated from the polymer fiber-reinforced paper. The demonstrated good comparison between the measured and the calculated load–displacement responses is considered, in this study, to adequately serve as the validation of the FE simulation processes.

**Figure 7:**
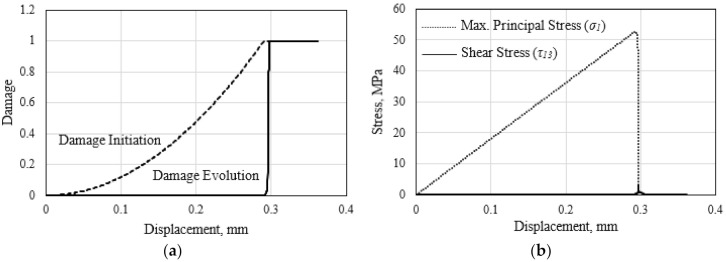
(**a**) Damage initiation and evolution to separation, and (**b**) the corresponding stresses at the critical material point of the cell wall of the HC core.

The two-stage material damage process at any critically stressed material point in the cell wall of the HC core panel is illustrated in Figure 7. The quadratic increase of the damage initiation variable for the tensile matrix damage follows Equation (3). Matrix damage initiates for this material point when the damage initiation variable, $d_{m}^{t}$, reaches the value of 1.0. This corresponds to the applied displacement of 0.29 mm, as illustrated in Figure 7a. Subsequent damage evolution is calculated based on Equation (5). When the damage evolution index, $d_{p}$ reaches the critical level, separation of the material point occurs. An increasing number of material points, particularly in the same vicinity, are expected to experience the state of separation with the continuously applied displacement. Collection of these separated material points are treated to form the traction-free crack surfaces.

In the out-of-plane tensile loading of the relatively brittle HC core material, the equivalent stress could be represented by the maximum principal stress, while the shear stress, τ_13_, is insignificant. The principal stress evolves linearly to reach the matrix tensile strength, *Y^T^*, of 51.8 MPa, at the onset of matrix cracking. Following the damage initiation event, the local stress decreases with the continuous loading, as shown in Figure 7b. It is noted that the damage evolution index, Equation (5), rises quickly to reach unity to indicate the separation of the material point. Simultaneously, the principal stress diminishes. It is worth mentioning that the calculated damage initiation index for fiber fracture, $d_{f}^{t}$, only reached a value of 0.21 at the end of the applied loading. Thus, matrix damage of the cell wall dominates the failure process.

**Figure 8:**
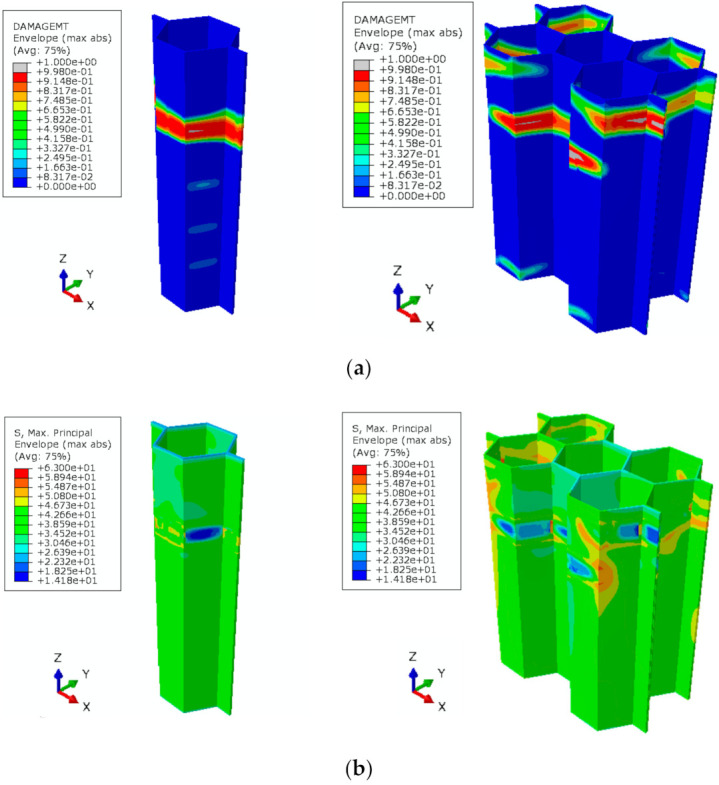
Contours of (**a**) damage and (**b**) maximum principal stress in the single-cell and four-cell HC core model. Values plotted correspond to matrix fracture of the cell wall, $d_{m}^{t}=0.998$.

**Figure 9:**
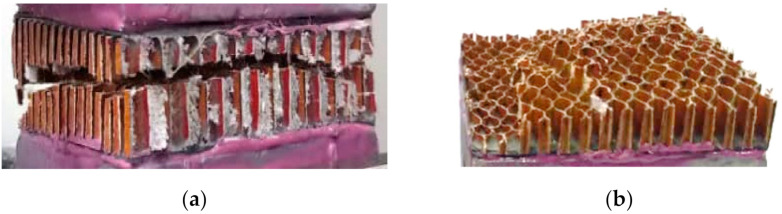
(**a**) Location of the fractured plane along the cell height, and (**b**) fractured surface of the polymer hexagonal HC core specimen, following the out-of-plane tensile loading.

The calculated distributions of the material damage due to the tensile matrix cracking in the single-cell and four-cell HC core model are compared in Figure 8a. The collection of the material points with the calculated damage index, $d_{p}$ is considered in this study to have formed a structural crack. Thus, the tensile loading conditions for Figure 8 have caused the formation of a crack in the cell wall of the HC core, as represented by the region with a damage index value above 0.998. It is observed that the critical section with the localized fracture is located closer to the displaced end of the model. A similar location of the fracture plane along the cell height has also been observed experimentally, as shown in Figure 9. It is worth noting that the crack initially forms at the single wall of the cell and propagate in Mode I towards the interconnected double-wall upon continuous loading. The corresponding stress distribution is illustrated in Figure 8b. The stress in the cell wall diminishes in the traction-free crack region. The equilibrium of the forces redistributes the stresses to localized in the critical section of the cell. Further tensile load increments would propagate the crack across the cells of the model. The similar distributions of the internal variables, including the matrix damage and the maximum principal stress, render the single-cell model to be sufficient to replicate the response of the HC core structure under the out-of-plane tensile loading.

### 3.2. Out-of-Plane Compressive Failure

**Figure 10:**
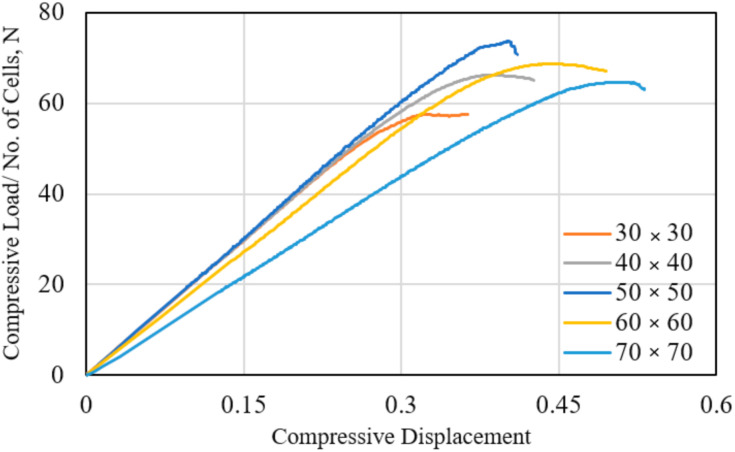
The measured compressive load–displacement responses of the HC core panel with a different cross-sectional area (cell size, *c* = 3.12; thickness, *H* = 12.7 mm).

The measured out-of-plane compressive responses of the Nomex polymer-based HC core panel of various square dimensions (core cell size, *c* = 3.2 mm and thickness, *H* = 12.7 mm) are shown in Figure 10. The load values are normalized by the number of cells in the respective sample. The experimental testing of these HC cores is done according to the procedure elaborated in the previous research work [45]. The compression tests are conducted in accordance with the ASTM C365 standard. Results show that the compressive stiffness could be adequately quantified for specimen size of up to 50 × 50 mm^2^. A larger specimen dimension is associated with a greater number of unconstrained walls along the perimeter, thus contributing to lower apparent stiffness. However, the smaller size specimen displays a lower apparent compressive strength of the HC core. This is likely due to the lower number of effective cells per unit area for the smaller specimen. The response of the 50 × 50 mm^2^ HC core panel is selected as the nominal load–displacement response of the material and used as the validation case for the FE model developed in this study.

**Figure 11:**
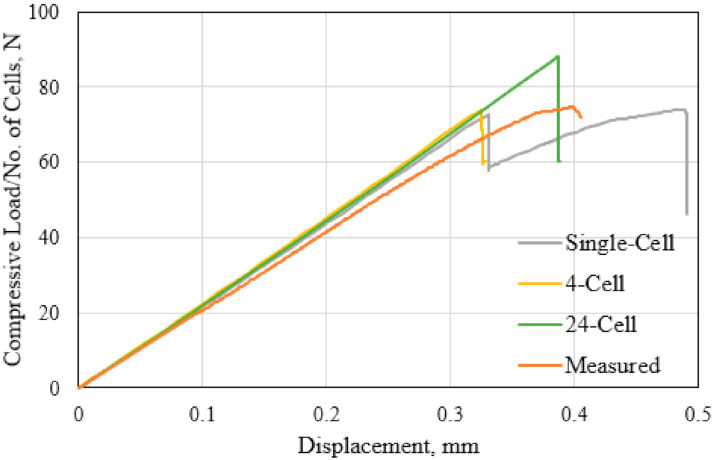
Comparison of the measured and the FE-calculated compressive load–displacement responses for the different FE models of the HC core.

The FE-calculated load–displacement responses of the polymer hexagonal HC core under the out-of-plane compressive loading, up to the onset of buckling, are compared with the measured curve, as shown in Figure 11. The sudden load drop from the peak level denotes the initiation of the crushing zone, as manifested in the localized buckling of the cell wall. The peak load defines the compressive strength of the respective HC core structure. The predicted compressive strength at 4.06±0.01 MPa by the single-cell and four-cell model is comparable to the measured strength of 4.01 MPa. A slightly higher (~4%) strength is calculated by the 24-cell model. The slight variation in the predicted onset of the buckling events is due to the localized influence of the unconstrained wall along the edges of the specimen. The measured out-of-plane compressive stiffness of the HC core is slightly lower (~3.2%) than the predicted value. This is likely due to the inhomogeneity of the HC cell wall, causing elastic buckling (wrinkling) and consequently results in slightly nonlinear responses prior to buckling of the specimen. A fairly good comparison of the measured and the FE-calculated load–displacement results are taken to adequately validate the FE simulation process.

**Figure 12:**
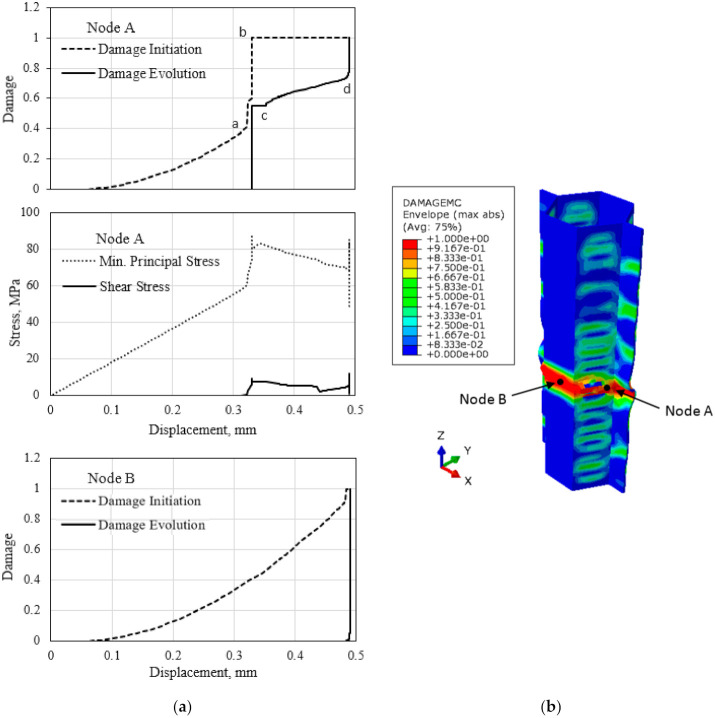
(**a**) Evolution of the matrix damage variable to the onset of damage and subsequent damage evolution, and the evolution of the (compressive) minimum principal stress. (**b**) Contour of the damage at the start of the global buckling of the HC cell.

The compressive buckling failure process of the cell walls of the polymer hexagonal HC core is described by using the single-cell model, as illustrated in Figure 12. The calculated damage variables to the onset of initiation and subsequent evolution to localized buckling event is shown in Figure 12a for node A and B. Node A is located in the single-wall closer to the unconstrained edges, while Node B is in the double-wall of the closed-cell. These nodes are critical with respect to the failure of the cell. Results show that the (compressive) minimum principal stress and the matrix damage initiation variable steadily increase with the applied load. A sudden “jump” of the matrix damage initiation variable, $d_{m}^{c}$ to the critical value of unity, corresponding to the applied displacement of 0.33 mm, indicates the onset of damage for the material point denoted as Node A. This also causes the matrix damage to surge (to $d_{p}$ = 0.55). The localized damage manifests in the observed global load drop. The damage continues to evolve while the stress decreases with the increasing applied load. At the applied displacement of 0.49 mm, the damage initiated and followed almost immediately to the critical value ($d_{p}$ = 1.0) at both locations. Other material points in the same section of the cell also experience the critical damage level, causing the observed global buckling of the cell structure. The additional applied displacement would cause the crushing and densification of the cell walls. The distribution of the matrix damaged in the HC cell wall corresponding to the start of the global buckling is illustrated in Figure 12b. It is worth mentioning that the identical location of the buckled section of the cell is observed in the compression test of the polymer hexagonal HC cell panel.

**Figure 13:**
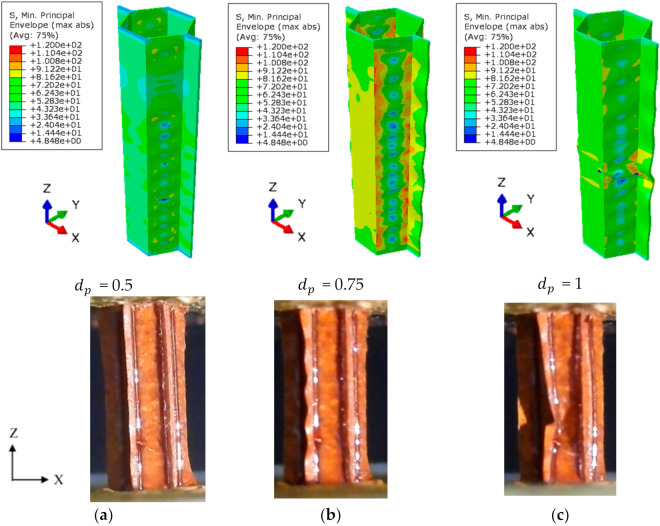
Distribution of the (compressive) minimum principal stress (top) and the deformation (bottom) during the out-of-plane compression test of the one-cell specimen. (**a**) Matrix damage, $d_{p}$ = 0, following damage initiation; (**b**) $d_{p}$ = 0.75, showing wrinkling of the unconstrained cell walls; and (**c**) $d_{p}$ = 1.0, with the occurrence of the first fold.

The typical distribution of the (compressive) minimum principal stress at the various stages of the damage evolution process in the single-cell polymer hexagonal HC core model is illustrated in Figure 13. The corresponding deformation of the one-cell specimen during the compression test is also shown. Stress localization is predicted to occur at different section planes in single walls of the single-cell model. The occurrence of the damage redistributes the stresses over the double walls of the model, as observed in Figure 13b for the damage index of $d_{p}$ = 0.75. This promotes elastic buckling in the form of wrinkling of the wall, as also observed experimentally. It is worth mentioning that similar stress distribution is predicted for all cells in the four-cell HC core model. Thus, the single-cell model adequately reproduces the localized buckling mechanism of the HC core panel based on the damage mechanics approach. The onset of localized buckling ($d_{p}$ = 1.0) occurs at the applied displacement of 0.49 mm, with the stress localizes in the vicinity of the buckled location. This also corresponds to the observed formation of the first folding of the unconstrained half-wall of the one-cell specimen, as shown in Figure 13c.

### 3.3. Out-of-Plane Shear Responses

**Figure 14:**
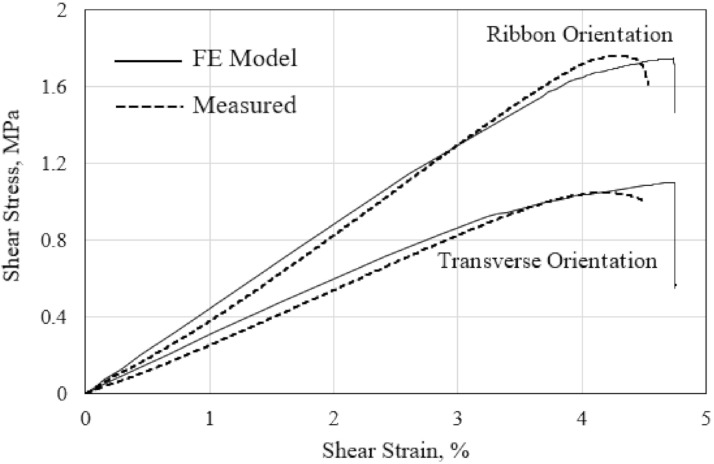
Comparison of the measured and FE-calculated out-of-plane shear stress–strain responses of the HC core.

The FE-calculated and measured responses of the polymer hexagonal HC core panel under the out-of-plane shear loading in the ribbon and transverse direction are compared in Figure 14. The measured response is taken from the out-of-plane shear test, performed in accordance with the ASTM C273 standard, as discussed in the previous research work [45]. The FE simulation of the out-of-plane shear behavior employs the 6-cell HC core model, as shown in Figure 2. The applied shear stress is defined as the shear force over the gross shear area of the HC core specimen or the six-cell HC core FE model, while the corresponding shear strain is calculated as the displacement per unit height of the specimen. The measured shear modulus of the polymer hexagonal HC core in the ribbon direction at 42.5 MPa is 59% higher than that in the transverse direction. Similarly, the measured shear strength is 60% higher in the ribbon direction as indicated by the peak of the curve at 1.68 MPa, compared to the strength in the transverse direction. This is likely due to the orientation of the double-wall of the HC cells parallel to the ribbon loading direction. The FE-predicted shear responses, with a maximum difference in stiffness of 9% and the shear strength of 3%, compared with the measured property, is fairly good. This is considering the scatter of the measured data, owing to the inherent inhomogeneity of the composite cell wall material and the variability of the thickness of the cell wall.

**Figure 15:**
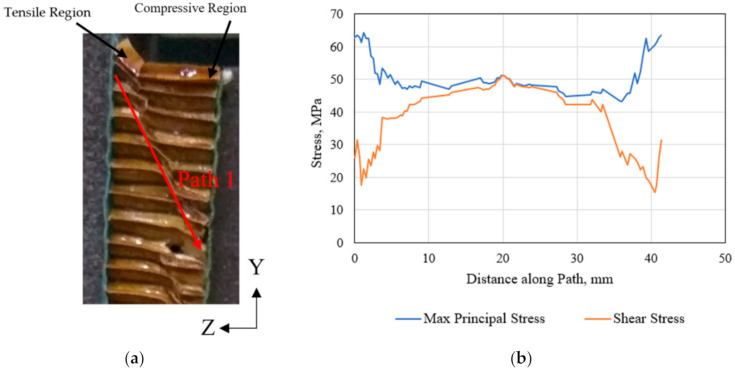
(**a**) Failed polymer hexagonal HC core specimen under the out-of-plane transverse shear, and (**b**) the corresponding FE-calculated principal stress and shear stress in the HC core model.

**Figure 16:**
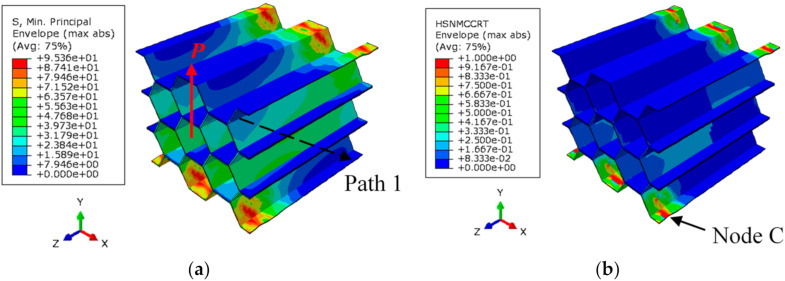
(**a**) Minimum principal (compressive) stress, and (**b**) matrix compressive damage contour, corresponding to the applied shear displacement of 0.33 mm.

The failed polymer hexagonal HC core specimen following the out-of-plane shear in the transverse direction [45] is shown in Figure 15a. Excessive deformation with buckling of the hexagonal cells is observed along the plane oriented at 45° to the applied shear loading direction (Path 1). The maximum normal stress is expected to occur on this critical plane. This is appropriately predicted by the representative six-cell model of the structure. The variation of the shear stress and the maximum principal stress in the shear test section (represented by Path 1 in Figure 15a and Figure 16a) is illustrated in Figure 15b. Results show that both types of stress are identical in magnitude in the mid-section plane of the specimen, thus ensuring that the pure shear test requirement prevails. Since both surfaces of the HC core specimen are bonded to the metal plate for applying the shear force, a tensile and compressive region is developed at the opposite edges of the specimen, as indicated in Figure 15a.

The distribution of the minimum (compressive) principal stress corresponding to the onset of induced localized buckling during the out-of-plane transverse shear test is shown in Figure 16a. Such high compressive stress magnitude contributes to the localized matrix damage in the edge region of the single-wall of the HC cell, as illustrated in Figure 16b. The compressive matrix damage represents the observed localized buckling of the cell wall (see Figure 15a). Similar distributions of the stress and damage variable are observed for the tensile counterpart.

**Figure 17:**
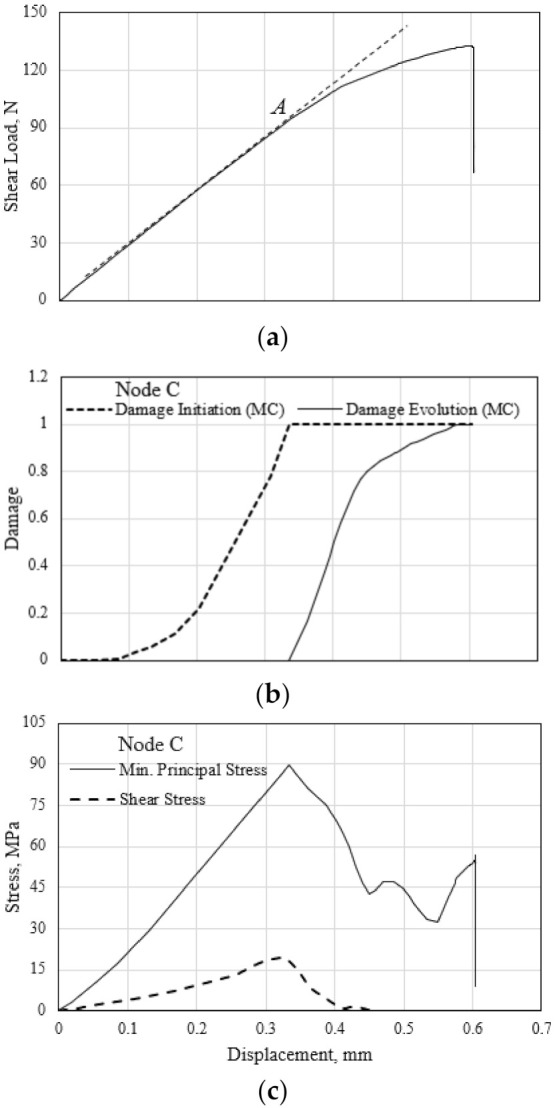
(**a**) Shear load–displacement response in the transverse direction. (**b**) Evolution of damage initiation variable and subsequent damage for the material point marked C in Figure 16b. (**c**) Evolution of stresses during the shear test of the HC core.

The shear load–displacement response of the polymer hexagonal HC core specimen in the transverse direction and the corresponding characteristic evolution of damage variables at the material point C (Figure 16b) of the HC cell is shown in Figure 17a,b, respectively. Results show that the onset of the compressive damage of the HC cell wall material, denoted by the damage initiation variable value of 1.0, is manifested in the observed initial deviation of the shear stiffness at the point marked A in Figure 17a. When the compressive matrix damage evolution variable reaches 1.0, localized buckling occurs for the material point C and similarly, for materials at the cross-opposite edges of the cell wall. This localized buckling failure contributes to the observed structural buckling of the HC cells, with the occurrence of the sudden shear load drop at the end of the test. Similar evolution characteristics are simultaneously displayed for the tensile matrix damage of the opposite edges of the HC core specimen. It is noted that no fiber damage in the critical cell wall is predicted throughout the out-of-plane shear test in the transverse direction.

The minimum principal (compressive) stress and the applied shear stress at Point C evolve during the out-of-plane shear in the transverse shear direction, as shown in Figure 17c. The stress increases with the monotonically applied shear force up to the peak value, corresponding to the initiation of the cell material damage. The damage evolves with continuous loading while the stress components diminish. The predicted fluctuation in the minimum principal stress beyond the applied shear displacement of 0.45 mm is caused by the localized wrinkling of the cell wall. Structural buckling of the HC cell occurs at the end of the test with the sudden drop in the stress magnitude.

**Figure 18:**
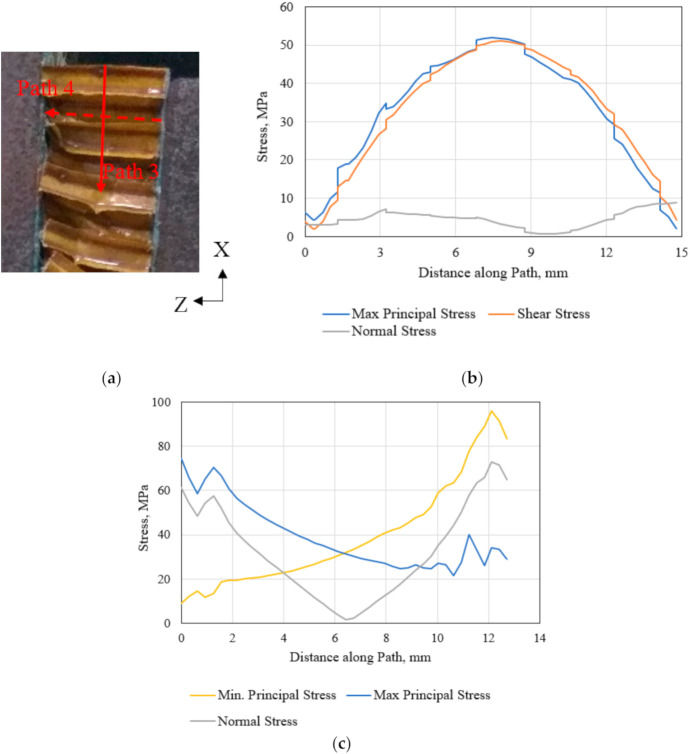
(**a**) Failed polymeric HC core specimen following the out-of-plane shear loading in the ribbon direction, (**b**) variation of the stresses along Path 3, and (**c**) Path 4 of the HC core model.

**Figure 19:**
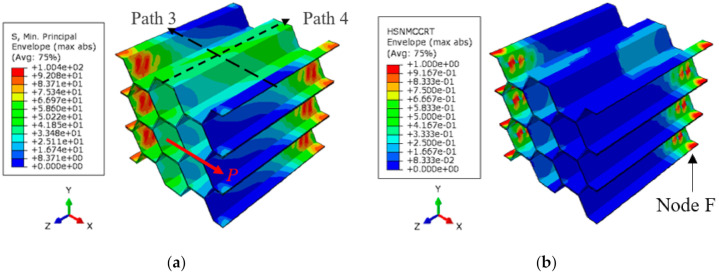
(**a**) Minimum principal (compressive) stress at failure, and (**b**) the corresponding compressive matrix damage of the HC core model, at the end of the shear test, in the ribbon direction.

The failed polymer hexagonal HC core specimen following the out-of-plane shear loading in the ribbon direction [45] is shown in Figure 18a. The variation of the shear and the maximum principal stress along the failed plane (Path 3) is similar, as illustrated in Figure 18b. This suggests the prevalence of the pure shear stress condition on the central plane of the failed specimen. Path 3 and Path 4 corresponding to the HC core specimen shown in Figure 18a are notified in the HC core FE model in Figure 19a. It is noted that the applied shear force induces tensile stress in one end and compressive stress in the other end of the critical HC cell, as illustrated in Figure 18c for Path 4. Such stress variation causes the observed localized flexure at the mid-section of the HC cells.

**Figure 20:**
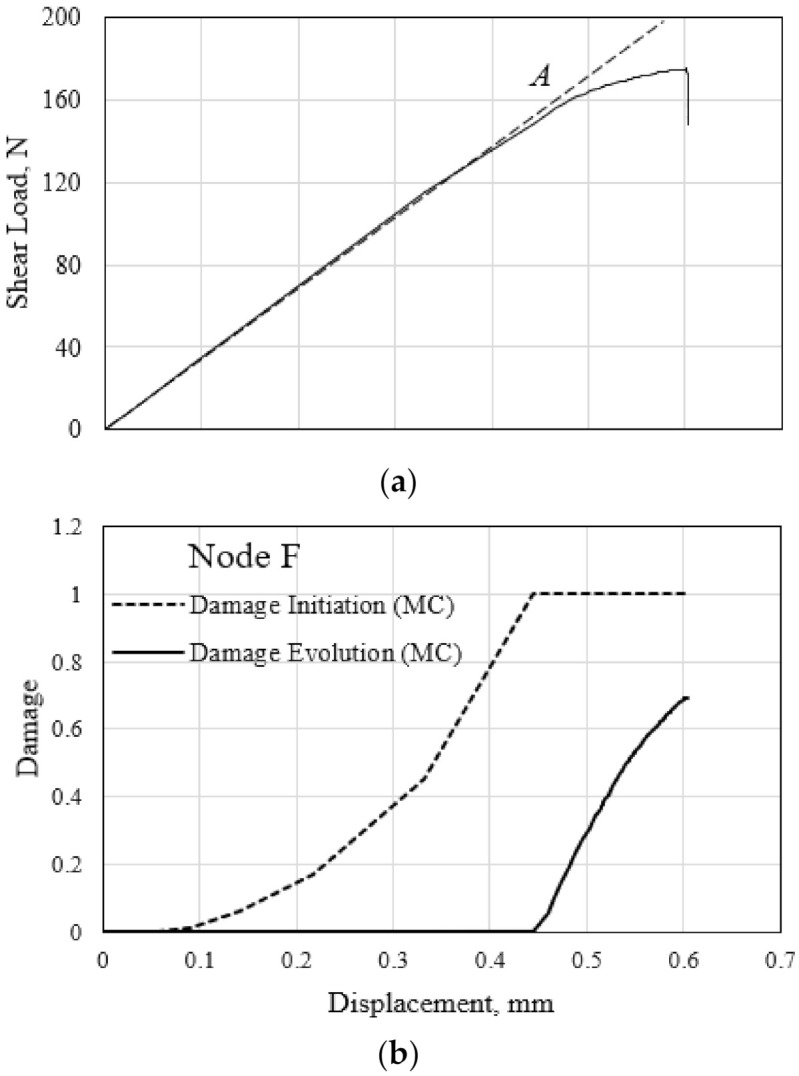
(**a**) Shear load–displacement response, and (**b**) characteristic evolution of the compressive matrix damage variables at the critical material point F.

The distribution of the minimum principal (compressive) stress at the failure of the polymer hexagonal HC core specimen is illustrated in Figure 19a. Opposite edges of the half-length walls of the cells experience the highest stress magnitude. This results in the accumulated compressive matrix damage of the materials in the form of localized buckling, as illustrated in Figure 19b. Similar distribution of the tensile stress and damage counterpart occurs in the opposite edges of the HC core specimen. The shear load–displacement and characteristic evolution of the compressive matrix damage at the critical material point F throughout the shear test is illustrated in Figure 20. The calculated damage initiation event at 0.45 mm is manifested in the onset of stiffness degradation of the polymer hexagonal HC core specimen, labeled A in Figure 20a. However, the subsequent damage evolution does not cause localized buckling or material yield in compression because the damage variable only attained a value of 0.7 at the end of the shear test in the ribbon direction. It is worth mentioning that the FE-calculated tensile matrix damage of the material point located at the opposite edge initiates earlier, at the applied shear displacement of 0.33 mm. Furthermore, the tensile matrix damage reaches a value of 1.0 at the end of the test, suggesting the fracture of the cell wall. This tensile-induced fracture is demonstrated in Figure 18a.

### 3.4. Summary of the Results

Different representative cell models of the hexagonal polymer HC core structure have been developed and examined. The smallest representative cell model consisting of 1 hexagonal cell (single-cell) for the tension and compression, and six cells (six-cell) for the out-of-plane shear loading could be used to predict deformation and failure response of structure. The boundary conditions for the symmetry of cell walls have been appropriately considered. These representative cell models employ the properties of the fiber-reinforced polymer cell wall material for the FE simulation [45]. In addition, the models with multiple cells (6- and 24-cell) are also examined for the tension and compression load case. The FE-predicted equivalent HC core properties are compared with measured values in Table 3. Although the calculated properties are comparable for the single and multiple cell models, it is noted that the single-cell model provides the closest to the measured property values for the tension and compression load case. The largest difference in elastic moduli, E_33_, and strength, σ_3_, is 5.1% and 2%, respectively, for the tension load case. The hexagonal six-cell models predicted the mechanical properties of the HC core with less than 9% difference from the measured value for the out-of-plane shear load cases. Thus, the smallest single-cell and six-cell model are appropriate to represent the mechanical responses of a large HC core panel for the respective out-of-plane loading conditions. It is worth noting that the single-cell model could not capture the crushing strength (i.e., the observed plateau stress) as the cell experiences the simultaneous crushing and densification due to the folding of the walls under compression. However, this could be alleviated using a model consisting of a group of “single-cells” to predict the crushing strength of the HC core structure.

**Figure 21:**
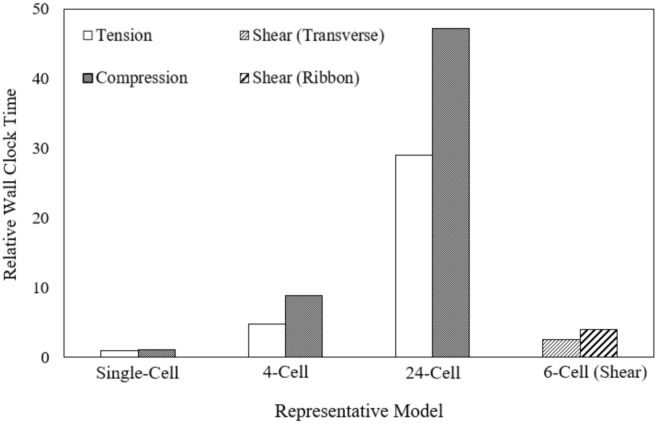
Comparison of relative wall-clock time taken by the different cell models FE models for the out-of-plane load cases.

The FE simulations are performed by using a desktop computer with four core processors. The FE models for all cases have an element edge length of 0.1 mm. The smallest representative cell model should be optimum with respect to the computational time of the simulation cases. The wall-clock time for the different cell models of the tension and compression load case is compared in Figure 21, relative to the single-cell tension load case. The relative wall-clock time for the shear load cases with the six-cell model are also compared. Results show that the wall-clock time increases exponentially, up to 30 and 48 times longer than that required for the single-cell model for the out-of-plane tension and compression load case, respectively. Although the smallest representative cell model for the shear load cases consists of six cells, the relative wall clock time is shorter than that for the four-cell model. This demonstrates the computational efficiency of the smallest single-cell model for the tension and compression, and six-cell model for the shear load cases.

## 4. Conclusions

The study has successfully identified the smallest representative single and multiple hexagonal cell models, to predict the response of the HC core structure under the out-of-plane loading conditions. The damage-based failure model of the Nomex polymer-based paper for the HC cell wall material enables the FE models to capture the observed reduction in the global stiffness and the associated load drop at fracture and/or buckling of the polymer hexagonal HC core structure. The following can be concluded:

- The single-cell FE model with continuum shell elements offers a closer prediction of the peak load to the measured tensile load level at fracture, compared to the model with the conventional shell elements. However, the 17.2% greater number of the unknown variables resulted in a 63.6% longer wall-clock time, rendering it computationally less efficient.
- The single-cell model is adequate in representing the out-of-plane tensile and compressive responses of the HC core structure with less than 5.0% difference in the measured stiffness and strength, compared with the 4-cell and 24-cell model.
- The smallest representative polymer hexagonal HC core model for the out-of-plane shear in the transverse and ribbon direction is a six-cell model. The predicted shear stiffness and load at the shear failure are within 9.0% and 3.0% variation, respectively, from the measured values.
- The onset of damage and the characteristic damage evolution of the critical cell wall material were shown to accurately manifest in the observed localized failure mechanism and the global stiffness degradation of the polymer hexagonal HC core structure.

The validated representative cell models could reduce the need for extensive experimental testing of the polymer hexagonal HC core structures, with different cell geometries, to quantify the structural properties. In addition, the models could serve for calibrating the predicted deformation responses through the failure of the equivalent homogenized model of the polymeric HC core structure. Such a homogenized model is employed in simulating the large and complex HC composite structures under the general loading conditions.

## Figures and Tables

**Table 1:** Properties of phenolic resin impregnated Nomex polymer-based paper.

Elastic Constants		Constitutive Damage Model Parameters	
$E_{11}$, (MPa)	3768	Longitudinal tensile strength*, X^T^* (MPa)	86.57
$E_{22}$, (MPa)	2879	Longitudinal compression strength, *X^C^* (MPa)	95.37
$E_{33}$, (MPa)	2647	Transverse tensile strength, *Y^T^* (MPa)	51.79
$G_{12}$, (MPa)	1367	Transverse compression strength, *Y^C^* (MPa)	78.5
$G_{13}$, (MPa)	1318	Longitudinal shear strength, *S^L^* (MPa)	82
$G_{23}$, (MPa)	1140	Transverse shear strength, *S^T^* (MPa)	40.7
*v_12_*	0.21	Longitudinal tensile fracture energy, *G_XT_* (N/mm)	2.18
*v_13_*	0.21	Longitudinal compression fracture energy, *G_XC_* (N/mm)	2.54
*v_23_*	0.21	Transverse tensile fracture energy, *G_YT_* (N/mm)	1.45
		Transverse compression fracture energy, *G_YC_* (N/mm)	2.17

**Table 2:** Comparison of the computational parameters used for the FE simulation of the single-cell model under tension with conventional and continuum shell elements.

Computational Variables	Shell Element Type	
	Conventional Shell (S4R)	Continuum Shell (SC8R)
Number. of elements	4662	5402
Number of nodes	4865	11,249
Total unknown variables (DOF ^1^)	29,190	34,206
Total CPU ^2^ time (s)	2554.4	3386
Wall clock time (s)	748	1224

^1^ Degree of freedom, ^2^ central processing unit.

**Table 3:** Comparison of the FE-predicted and measured property values of the polymer HC core structure under the out-of-plane loadings.

		Elastic Moduli (MPa)		Strength (MPa)	
Load Case		FE Model	Measured	FE Model	Measured
Tension	Single-cell	144.5	137.5	3.4	3.47
	4-cell	147.2		3.32	
	24-cell	147.6		3.38	
Compression	Single-cell	142.7	140.74	4.06	4.01
	4-cell	143.8		4.06	
	24-cell	144.5		4.27	
Shear (transverse)	6-cell	29.3	25.07	1.12	1.09
Shear (ribbon)	6-cell	44.5	42.5	1.65	1.68

## Appendix A

The elastic constants ($E_{11}$, $E_{22}$), tensile strength (*X^T^*, *Y^T^*), and the respective fracture energies (*G_XT_*, *G_YT_*) of the phenolic resin impregnated polymer Nomex paper are calculated from the stress–strain plots, as shown in Figure A1. The remaining elastic constants and damage model parameters are estimated based on the ratio of the elastic constant, $E_{11}$, in the current and previous research work [20].
